# Social networking and fear of missing out (FOMO) among medical students at University of Khartoum, Sudan 2021

**DOI:** 10.1186/s40359-023-01403-z

**Published:** 2023-12-01

**Authors:** Abdalla Abbas Abdalla Mohammed, Mohammedahmed M. Osman, Mohammednour Mukhtar Mohammednour Ali, Mohammed O. Adam, Mohamed Esameldeen Elsayed Mustafa, Anab M. Babikir, Ahmed M. I. Abdulsamad, Mohamed Elhadi Abdelrahim Abo, Yasein Abdelmoneim Mohamed Yasein, Mohamed Sati Shampool Abdelgader, Elfatih A. Hasabo, Mohammed Elsir Ibrahim

**Affiliations:** Faculty of Medicine, University of Khartoum, ElQasr Avenue, Khartoum, Khartoum State 11111 Sudan

**Keywords:** Social networking sites, Social networking intensity, Fear of missing out

## Abstract

**Background:**

With students becoming more involved in the internet and social networking sites, they become more prone to their consequences. This study focuses on measuring the social networking intensity and the fear of missing out among the medical students of University of Khartoum, then examining the association between them.

**Methods:**

Facility-based, descriptive, cross-sectional study was conducted at Faculty of Medicine, Khartoum University between January and March 2021. A total of 333 students were selected by simple random sampling. Data was collected from the participants using a structured self-administered questionnaire that involved the social networking intensity (SNI) scale and fear of missing out (FOMO) scale. The data was analyzed by the Statistical Package for Social Science (SPSS) software version 26.

**Results:**

Moderate positive correlation between social networking intensity and fear of missing out was found (*p*-value < 0.01). Of the total participants; 51 participants (15.4%) experienced low SNI and low FOMO. Another 78 participants (23.6%) had moderate SNI and moderate FOMO and only 16 Participants (4.8%) showed high SNI and high FOMO. There were no significant differences in SNI or FOMO scores among the different socio-demographic variables, except for the SNI score which was positively correlated to the monthly income.

**Conclusion:**

An association between SNI and FOMO was found. SNI was not affected by socio-demographic factors except for the monthly income.

**Supplementary Information:**

The online version contains supplementary material available at 10.1186/s40359-023-01403-z.

## Introduction

### Background

The twenty-first century has witnessed a worldwide spread of digital devices that have opened the doors for many people to communicate with each other. With a global population of 7.83 billion at the start of 2021, we find that 5.22 billion people use a mobile phone and this equals 66.6% of the world’s total population. Of those: 4.20 billion are social media users; this constitutes more than 53% of the world’s total population [1]. Mobile phones, the internet, and social networking sites (SNSs) can be useful or harmful; they revolutionized human communication in unprecedented way and provided a stimulus for progress in data science [2]. But if used excessively, social networking sites can have many bad consequences [3, 4]; research in this field have led to the introduction of new terms like; Problematic Smartphone Use (PSU), Problematic Mobile phone Use (PMPU), Internet Addiction (IA), Problematic Internet Use (PIU), Social Networking Sites (SNSs), Social Networking Intensity (SNI) [5–8]. Social networking is defined here as using dedicated, internet-based websites and apps to interact with other users; this is a broad definition and it includes popular social media services as well as other sites such as e-mail services and forums.

One of the latest problems that have been associated with heavy social networking is fear of missing out (FOMO), which is characterized by a strong desire to remain in touch with what others are doing, acting like a driving force behind social media use [9, 10]. Moreover, excessive social networking is related to many important psychological problems such as depression and anxiety, and fear of missing out (FOMO) is regarded as a mediator for these problems [11]. FOMO is considered one of the challenges of today life and it has been connected to problematic social media engagement. People -especially young adults- use social media at inappropriate times; during meetings, during classes, and while driving so FOMO is considered to be related to increased number of accidents [9, 12, 13]. These negative consequences of heavy social networking and fear of missing out are usually underestimated or overlooked. Many studies have been conducted to assess intensive social networking and its association with Internet Addiction (IA), Problematic Internet Use (PIU), and psychological disturbances. But published studies on fear of missing out is limited and more effort is required to deal with this problem. Moreover, less work has been done to assess the correlation between intensive social networking and fear of missing out, and most of the published papers are more relevant to communities with high to moderate socio-economic status which comprise further limitation on data about FOMO and its role with social networking in our country.

FOMO is a controllable problem, but it doesn’t get enough attention. This research is expected to draw more attention towards it and its relation to SNI among psychologists, behaviorists, caregivers, and parents to put strategies that can help in dealing with those issues.

The aim of this study is to assess the social networking intensity, fear of missing out, and the relationship between them among medical students at the university of Khartoum, Sudan.

## Methodology

### Study design and setting

This is an institution-based, descriptive, cross-sectional study. It was conducted at the Faculty of Medicine, University of Khartoum, Khartoum state, Republic of Sudan in January, February, and March 2021.

We included all undergraduate medical students at the faculty of medicine, university of Khartoum (the total population was 2016 students from 6 classes, both males and females). We excluded students unwilling to participate.

### Sampling

With a total population (N) of 2016 and a level of precision (d) of 0.05; a sample size (n) of 333 students was calculated using the formula: $n=N/1+(N\times d^2 )$. The sample was selected using proportionate simple random sampling.

The total population was divided into 6 classes that correspond to the academic years. The size of the student sample from each class was proportionate to the size of the class (56 from 1^st^ year class, 54 from 2^nd^ year class, 55 from 3^rd^ year class, 59 from 4^th^ year class, 57 from 5^th^ year class, 52 from 6^th^ year class). Students from each class were selected by simple random sampling.

Data was collected using an online, self-administered structured questionnaire (Google form) which consisted of sociodemographic data, social networking intensity (SNI) scale, and fear of missing out (FOMO) scale. Names were not included to ensure confidentiality.

The questionnaire (Supplementary 1) was developed for this study and composed of 23 items, divided into 3 sections. The socio-demographic characteristics section was composed of 6 variables (Age—Sex—Batch—Place of residence—Marital status—Monthly income) (Table 1). Participants were asked about the way they access the internet through most of the time (smartphones or laptops), and how they connect to the internet (Table 1). In social networking intensity section, a scale of 5 items was used to assess the level of social networking intensity (SNI) of each participant, each item used a 5-point Likert scale (1 = ‘‘Not at all true for me’’, 2 = ‘‘Slightly true for me’’, 3 = ‘‘Moderately true for me’’, 4 = ‘‘Very true for me’’, and 5 = ‘‘Extremely true for me’’), SNI score for each individual was calculated by summation of the five items. Scores ranged between 5 and 25 (5 represents low SNI and 25 represents the highest level of SNI). According to Salehan and Negahban, this scale has a good internal consistency, with a Cronbach alpha coefficient of 0.88 [8]. In our study, the Cronbach alpha coefficient for the SNI scale was 0.84 which suggests a good internal consistency and reliability for the scale regarding our sample (Table 2). SNI scores were classified into three grades: low (scores 5–10), moderate (scores 11–19), and high (scores 20–25).

**Table 1:** Socio-demographic characteristics and internet access

**Sex**	N	%
Males	136	41.1%
Females	195	58.9%
**Age**		
16_20	109	32.9%
21_25	217	65.6%
26_30	5	1.5%
**Residence**		
With family	247	74.6%
Dormitory (university campus)	71	21.5%
With relatives	13	3.9%
**Marital status**		
Single	319	96.4%
Engaged	6	1.8%
Married	6	1.8%
**Monthly income**		
10,000 _ 20,000 SDG	153	46.2%
20,001 _ 30,000 SDG	62	18.7%
30,001 _ 40,000 SDG	37	11.2%
40,001 _ 50,000 SDG	79	23.9%
**Batch**		
92	52	15.7%
93	57	17.2%
94	60	18.1%
95	54	16.3%
96	54	16.3%
97	54	16.3%
**Access to internet**		
Using phone	328	99.1%
Using laptop	3	00.9%
**Access to internet**		
Using my own data bundle	289	87.3%
Using family WIFI	042	12.7%

**Table 2:** Social networking intensity scale

	Not at all true of me N %		Slightly true of me N %		Moderately true of me N %		Very true of me N %		Extremely true of me N %	
Visiting social networking sites is part of my everyday activity.	13	3.9%	38	11.5%	67	20.2%	72	21.8%	**141**	**42.6%**
I check my social networking site(s) almost every day.	16	4.8%	38	11.5%	53	16.0%	76	23.0%	**148**	**44.7%**
I feel out of touch when I have not logged onto my social networking site(s) for a day.	**89**	**26.9%**	78	23.6%	58	17.5%	43	13.0%	63	19.0%
I feel I am part of the community of my social networking site on campus.	**121**	**36.6%**	90	27.2%	60	18.1%	31	9.4%	29	8.8%
I would be sorry if my social networking site shuts down.	69	20.8%	65	19.6%	54	16.3%	**75**	**22.7%**	68	20.5%

In fear of missing out section, a scale of 10 items was used to assess FOMO among the participants, each item used a 5-point Likert scale (1 = ‘‘Not at all true for me’’, 2 = ‘‘Slightly true for me’’, 3 = ‘‘Moderately true for me’’, 4 = ‘‘Very true for me’’, and 5 = ‘‘Extremely true for me’’), FOMO score for each participant was calculated by summation of the ten items. Scores ranged between 10 and 50 (10 represents low FOMO and 50 represents the highest level of FOMO). According to Przybylski, Murayama, DeHann, and GladWell (2013), this scale has a good internal consistency, with a Cronbach alpha coefficient of 0.89 [9]. In our study; we had a Cronbarch alpha coefficient of 0.88 which suggests reliable results with good internal consistency for the FOMO scale regarding our sample (Table 3). FOMO scores were classified into three grades: low (scores 10–20), moderate (scores 21–39) and high (scores 40–50).

**Table 3:** Fear of missing out on the scale

	**Not at all true of me N %**		**Slightly true of me N %**		**Moderately true of me N %**		**Very true of me N %**		**Extremely true of me N %**	
I fear others have more rewarding experiences than me	**179**	**54.1%**	66	19.9%	40	12.1%	28	8.5%	18	5.4%
I fear my friends have more rewarding experiences than me	**177**	**53.5%**	74	22.4%	32	9.7%	26	7.9%	22	6.6%
I get worried when I find out my friends are having fun without me	**118**	**35.6%**	105	31.7%	48	14.5%	31	9.4%	29	8.8%
I get anxious when I don't know what my friends are up to	**182**	**55.0%**	68	20.5%	41	12.4%	21	6.3%	19	5.7%
I must understand my friend's in-jokes	**108**	**32.6%**	81	24.5%	66	19.9%	41	12.4%	35	10.6%
Sometimes, I wonder if I spend too much time keeping up with what is going on	66	19.9%	75	22.7%	73	22.1%	52	15.7%	65	19.6%
It bothers me when I miss an opportunity to meet up with friends	82	24.8%	99	29.9%	68	20.5%	49	14.8%	33	10.0%
When I have a good time I need to share the details online (e.g. updating status)	**198**	**59.8%**	76	23.0%	36	10.9%	6	1.8%	15	4.5%
When I miss out on a planned get-together it bothers me	76	23.0%	90	27.2%	73	22.1%	55	16.6%	37	11.2%
When I go on vacation, I continue to keep tabs on what my friends are doing	**117**	**35.3%**	93	28.1%	56	16.9%	38	11.5%	27	8.2%

### Data management and analysis

Statistical Package for Social Science 26 (SPSS-26) software was used for data entry and analysis. Simple descriptive statistics were used to determine the frequencies and percentages of the different variables. Cronbach's alpha coefficient was calculated for the two scales to determine their internal consistency. Pearson correlation coefficient was used to assess the association between SNI & FOMO. Linear regression analysis was used to describe the relation between FOMO and SNI.

Independent t-test and one-way ANOVA were used to examine the associations and differences related to the socio-demographic groups.

## Results

### Sociodemographic characteristics

Most of our participants were between 21 and 25 years old (65%), with almost 60% of them being females and 40% males. Other sociodemographic characteristics are shown in Table 1. Regarding access to the internet; almost all of the participants (99.1%) used mobile phones rather than laptops to access the internet, and most of them (87.3%) used their data bundles compared to (12.3%) who used home Wifi (Table 1).

### Social networking intensity (SNI) score (Table 4)

**Table 4:** Social networking intensity and fear of missing out on scores

**Social networking intensity (SNI) Grades**	N	%
Low (scores 5–10)	62	18.7%
Moderate (scores 11–19)	176	53.2%
High (scores 20–25)	93	28.1%
**FOMO Grades**	N	%
Low (scores 10–20)	164	49.5%
Moderate (scores 21–39)	145	43.8%
High (scores 40–50)	22	6.6%

Almost half the sample (53.2%) had moderate SNI grades (scored 11–19), and nearly one-third (28.1%) had high SNI grades (scored 20–25).

### Regarding the fear of missing out (FOMO) score (Table 4)

43.8% of the participants showed moderate levels of FOMO, with 6.6% having high FOMO scores.

### Correlation between social networking intensity (SNI) and fear of missing out (FOMO)

**Fig. 1:**
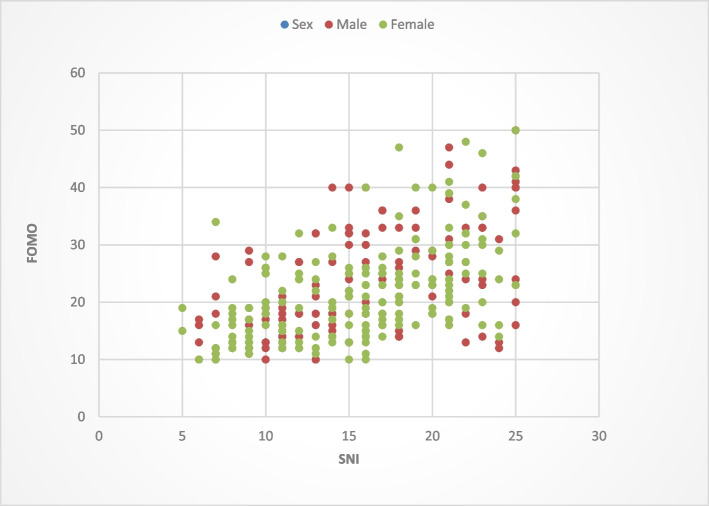
Grouped scatterplot of FOMO by SNI and sex

Of the total participants; 51 participants (15.4%) had low SNI and low FOMO, 78 participants (23.6%) had moderate SNI and moderate FOMO and 16 Participants (4.8%) had high SNI and high FOMO. A scatterplot of FOMO score by SNI score and sex is shown in Fig. 1.

**Fig. 2:**
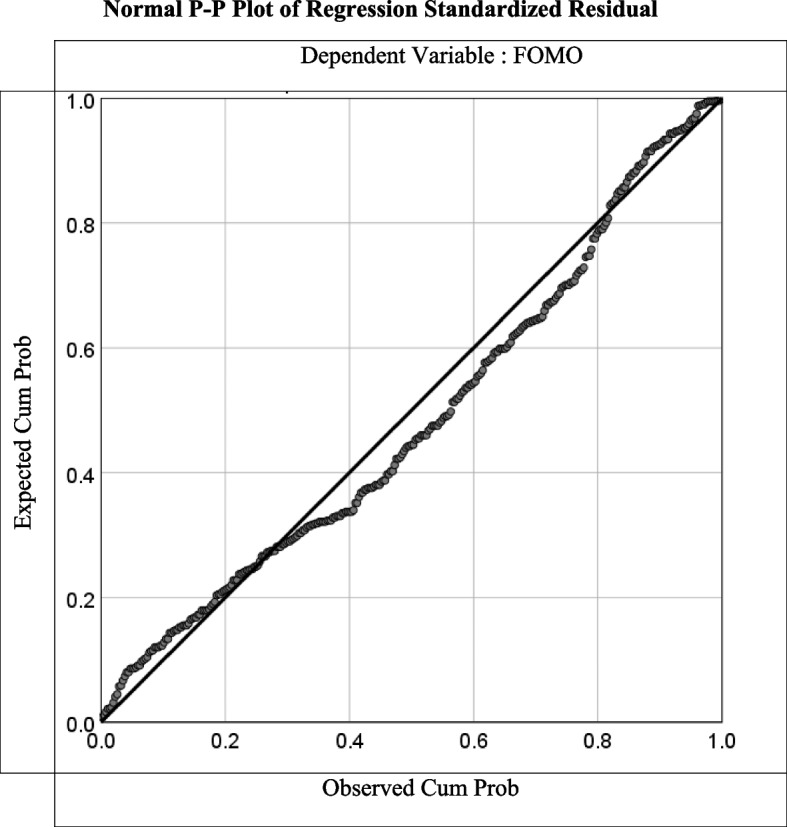
Normal P-P plot of regression

The results show a moderate positive correlation between social networking intensity and fear of missing out (*r* = 0.53, *p*-value < 0.001) (significant *p*-value is ≤ 0.05), (Table 5). By linear regression analysis, the relation between FOMO and SNI scores is described by the equation: $\mathrm Y\;=\;8.188\;+\;0.904\;\times\;\left(\mathrm X\;=\;\mathrm{Intensity},\;\mathrm Y=\;\mathrm{FOMO}\right)$, it’s plotted in Fig. 2.

**Table 5:** Pearson correlation between SNI and FOMO

		**Social Networking Intensity (SNI)**
**Fear Of Missing Out (FOMO)**	**Pearson Correlation**	.533**
	**Sig. (2-tailed)**	**< .001**
	**N**	331

^**^Correlation is significant at the 0.01 level (2-tailed)

### Association between different variables and SNI and FOMO scores

By independent sample t-tests, no significant difference in the mean SNI and FOMO scores was found with regard to sex, internet access, and way of connection (Table 6).

**Table 6:** *P* values from independent sample t and ANOVA tests for difference in the mean scores of SNI and FOMO among different groups

***p* values from independent sample t-test**	**SNI**	**FOMO**
Sex (male, female)	0.948	0.094
Internet access (smartphones, laptops)	0.40	0.249
Connection to the internet (family WIFI, data bundles)	0.573	0.483
***P* values from one-way ANOVA**	**SNI**	**FOMO**
Age	0.576	0.265
Batch	0.373	0.158
Residence	0.510	0.164
Marital status	0.155	0.101
Monthly income	0.05*	0.662

^*^Significant *p*-value is ≤ 0.05

One-way between-groups analysis of variance (ANOVA) was conducted to explore the impact of socio-demographic variables on SNI and FOMO scores. The results show no significant difference in SNI and FOMO scores with regard to the age groups, residences, batches, and marital statuses. However, the results show a significant difference among the four groups of monthly income with regard to the social networking intensity score *F* (3, 327) = 2.575, (*p*-value = 0.05). Despite being statistically significant, the actual difference in mean score between the groups is quite small. The effect size, calculated using eta squared, is (0.02). Post-hoc analysis using the Tukey HSD test indicates that the mean score for Group 2 (*M* = 14.50, *SD* = 5.44) was significantly different from Group 4 (*M* = 16.89, *SD* = 5.05). On the other hand, there is no statistically significant difference in FOMO scores among the four groups (*p* = 0.66) (Table 6).

## Discussion

FOMO can be considered as a form of behavioral addiction, so it doesn’t come by surprise that this study found that there is a moderate positive correlation between social networking intensity (SNI) and fear of missing out (FOMO). This finding is also consistent with a number of studies done in countries with different socio-demographic backgrounds [12–15]. Besides providing an additional evidence for this issue, it’s also fascinating to see how similar social phenomena can arise almost simultaneously in countries so far and so different, this reflects the effect of massive spread of digital devices around the world. The results don’t specify the direction of this relationship, but it’s likely that both SNI and FOMO affect each other; the more intense social networking is, the higher the level of compulsion due to FOMO becomes and vice versa. It should be noted that this relationship is not as linear as this statement implies; human behavior is complex and is affected by numerous factors, that’s why some participants had a high SNI score but a low FOMO score. This doesn’t abolish the relationship between them, but a better understanding of the factors that can have an influence on this relation might come in handy when dealing with it.

There was no significant difference in SNI and FOMO scores between males and females. With regard to the main issue here (FOMO), a study done in Bosnia supports this finding [13], but there are studies that found a gender difference with some reporting the highest levels of FOMO in females [16] and others in males [9]. It’s difficult to make sense of this apparent contradiction; the latter cited study had a wider age group with significantly larger mean (43.21, compared to 13 and 14.87 respectively), this may partially explain the results. It is also possible that cultural gender differences play a role. And in the end, mere chance remains an explanation for this difference.

Regarding age, our study didn’t show a significant difference in SNI and FOMO scores. This supported by a study done in Belgium [16] but is opposed by other studies that found a negative correlation between age and both FOMO and social media engagement [9, 12]. The wider age group of the latter two cited studies possibly explains why they had results that differ from our study; younger individuals are more likely to use social networking services frequently and thus are more prone to its effects including FOMO, and since our participants comprise a narrow age group and limited to a younger age it may not show the same variability as the mentioned studies.

There was also no significant difference in SNI and FOMO scores regarding the other socio-demographic variables assessed in this study (age, batch, marital status, residence, monthly income), except for SNI scores being higher with higher monthly income. This association between SNI and monthly income is supported by the findings of Bosnia study [13]. This is probably related to the fact that most of the participants (87%) use their mobile data because more financially capable individuals can afford the cost of better internet plans and thus have more access to social networking services.

The finding that FOMO score was generally uniform among the various socio-demographic groups worth some attention; in behavioral studies it’s common to find some variation amongst different groups. It’s difficult to draw a conclusion based on this study alone; but having a factor that can influence different groups to the same extent says a lot about its strength and so it deserves more study and attention.

## Limitations

Our study had several limitations that should be considered while interpreting the findings. Firstly, We didn’t assess the association between different social networking sites (Facebook, Twitter…) individually and fear of missing out. Secondly, we didn’t assess social networking intensity by hours of mobile usage in social networking sites, it wasn’t included in the SNI scale for it may compromise the validity of the score because of its relation to a number of factors that can be confounding (e.g., free time, internet availability…). Finally, monthly income cutoff points that we have used possibly don’t accurately reflect the level of socioeconomic status.

## Conclusion

This study is the first one in Sudan that explores the FOMO phenomenon and its association with SNI. A moderate positive correlation between social networking intensity and fear of missing out was found. This finding doesn’t only demonstrate the important relationship between SNI and FOMO, but it also sheds light on the effect of social media on human thinking and behavior that have become widespread and even reached the poorer countries.

## Recommendations

Awareness campaigns and educational sessions must be implemented to warn the students about these emerging problems. We recommend that problems associated with internet use (PIU, PSU, PMPU, SNI & FOMO) must be included in the curriculum (in psychology and psychiatry courses) as they result in many negative consequences. Further research in Sudan must be done to bridge the knowledge gap. As this is the first study, its limitations are of great concern and more reliable questionnaire is needed which excludes the psychological problems that may result in FOMO and assesses the activities and hobbies that may help in reducing FOMO or act as protecting factors.

### Supplementary Information

**Additional file 1.** Questionnaire.

## Data Availability

The datasets used and/or analyzed during the current study are available from the corresponding author on reasonable request.

## References

[1] Kemp S. Digital 2021: Global Overview Report — DataReportal – Global Digital Insights [Internet]. Hootsuite & We Are Social. 2021 . Available from: https://datareportal.com/reports/digital-2021-global-overview-report. Cited 2021 Feb 26.

[2] Dwivedi YK, Kelly G, Janssen M, Rana NP, Slade EL, Clement M (2018). Social Media: The Good, the Bad, and the Ugly. InformSyst Front.

[3] Oberst U, Wegmann E, Stodt B, Brand M, Chamarro A (2017). Negative consequences from heavy social networking inadolescents: The mediating role of fear of missing out. J Adolesc.

[4] Vaidya N, Jaiganesh S, Krishnan J (2016). Prevalence of internet addiction and its impact on the physiological balance of mental health. Natl J Physiol Pharm Pharmacol.

[5] Elhai JD, McKay D, Yang H, Minaya C, Montag C, Asmundson GJG. Health anxiety related to problematic smartphone use and gaming disorder severity during COVID-19: Fear of missing out as a mediator. Hum Behav Emerg Technol. 2021;3(1):137–46. 10.1002/hbe2.227. 10.1002/hbe2.227 PMC7753448 33363275

[6] Coskun S, KarayagızMuslu G (2019). Investigation of Problematic Mobile Phones Use and Fear of Missing Out (FoMO) Level in Adolescents. Community Ment Health J.

[7] Boyd DM, Ellison NB (2007). Social network sites: definition, history, and scholarship. J Comput Commun.

[8] Salehan M, Negahban A (2013). Social networking on smartphones: When mobile phones become addictive. Comput Human Behav.

[9] Przybylski AK, Murayama K, Dehaan CR, Gladwell V (2013). Motivational, emotional, and behavioral correlates of fear of missing out. Comput Human Behav.

[10] Can G, Satici SA (2019). Adaptation of fear of missing out scale (FoMOs): Turkish version validity and reliability study. Psicol Reflex e Crit..

[11] Elhai JD, Yang H, Montag C (2020). Fear of missing out (FOMO): overview, theoretical underpinnings, and literature review on relations with severity of negative affectivity and problematic technology use. Brazilian J Psychiatry.

[12] Chamarro LA. Fear of Missing Out, online social networking and mobile phone addiction: a latent profile approach. Aloma Rev Psicol ciències l’educació i l’esport Blanquerna. 2017;35(1):22–30.

[13] Tomczyk Ł, Selmanagic-Lizde E (2018). Fear of Missing Out (FOMO) among youth in Bosnia and Herzegovina — Scale and selected mechanisms. Child Youth Serv Rev.

[14] Traş Z, Öztemel K (2019). Examining the relationships between Facebook intensity, fear of missing out, and smartphone addiction. Addicta Turkish J Addict.

[15] Wolniewicz CA, Tiamiyu MF, Weeks JW, Elhai JD (2018). Problematic smartphone use and relations with negative affect, fear of missing out, and fear of negative and positive evaluation. Psychiatry Res.

[16] van Rooij AJ, Lo Coco G, De Marez L, Franchina V, Van den Abeele M (2018). Fear of missing out as a predictor of problematic social media use and phubbing behavior among flemish adolescents. Int J Environ Res Public Health..

