# Supplementary material for: Social networking and fear of missing out (FOMO) among medical students at University of Khartoum, Sudan 2021
Source: BMC Psychol. 2023 Dec 1;11:422. doi: 10.1186/s40359-023-01403-z (PMC10693061; doi:10.1186/s40359-023-01403-z)
Supplement: Supplementary file 1 — Additional file 1. Questionnaire. [file 40359_2023_1403_MOESM1_ESM.docx]

**Questionnaire:**

This questionnaire is about social networking intensity and its association with fear of missing out. FOMO was defined as a pervasive apprehension that others might be having rewarding experience from which one is absent .and it is characterized by the desire to stay continually connected with what others are doing. It is a kind of driving force behind social media use (one of the main reasons that keep people online) and although people use the social media to be connected to others, the social networking that is derived by FOMO may end up with loneliness and the feel of disconnection as a result of becoming connected to the virtual community without any real contact with others.

Sex

Male

Female

Age

11 _ 15

16 _ 20

21 _ 25

26 _ 30

Batch

92

93

94

95

96

97

Place of residence

With my family

With my relatives

Dormitory (University residence)

Marital status

Single

Engaged

Married

Divorced

Monthly income

10 000 _ 20 000 SDG

20 001 _ 30 000 SDG

30 001 _ 40 000 SDG

40 001 _ 50 000 SDG

How do you access the internet most of the times?

Using my phone

Using a laptop

Using a computer

Internet café

I don’t have a mean to reach it.

How do you connect to the internet most of the times?

Using my own data

Using the family WIFI

Through the university/dormitory WIFI

I can’t connect to the internet

Below is a collection of statements about your everyday activities and experiences. Using the scale provided please indicate how true each statement is of your general experiences. Please answer according to what really reflects your experiences rather than what you think your experiences should be. Please treat each item separately from every other item.

Response anchors;

Not at all true of me (1 )

Slightly true of me ( 2 )

Moderately true of me ( 3 )

Very true of me ( 4 )

Extremely true of me ( 5 )

Social Networking Intensity Scale (SNI)

|  | 1 | 2 | 3 | 4 | 5 |
| --- | --- | --- | --- | --- | --- |
| Visiting social networking sites is part of my everyday activity. |  |  |  |  |  |
| I check my social networking site(s) almost every day |  |  |  |  |  |
| I feel out of touch when I have not logged onto my social networking site(s) for a day |  |  |  |  |  |
| I feel I am part of the community of my social networking site at the campus |  |  |  |  |  |
| I would be sorry if my social networking site shuts down |  |  |  |  |  |

Fear Of Missing Out Scale (FOMO)

|  | 1 | 2 | 3 | 4 | 5 |
| --- | --- | --- | --- | --- | --- |
| I fear others have more rewarding experiences than me. |  |  |  |  |  |
| I fear my friends have more rewarding experiences than me. |  |  |  |  |  |
| I get worried when I find out my friends are having fun without me. |  |  |  |  |  |
| I get anxious when I don't know what my friends are up to. |  |  |  |  |  |
| It is important that I understand my friends "in jokes." |  |  |  |  |  |
| Sometimes, I wonder if I spend too much time keeping up with what is going on. |  |  |  |  |  |
| It bothers me when I miss an opportunity to meet up with friends. |  |  |  |  |  |
| When I have a good time it is important for me to share the details online (e.g. updating status). |  |  |  |  |  |
| When I miss out on a planned get-together it bothers me. |  |  |  |  |  |
| When I go on vacation, I continue to keep tabs on what my friends are doing. |  |  |  |  |  |

**Medical campus, University of Khartoum**
